# Global food and farming futures

**DOI:** 10.1098/rstb.2010.0181

**Published:** 2010-09-27

**Authors:** John Beddington

**Affiliations:** Chief Scientific Adviser to the UK Government, Head of the Government Office for Science, UK

Food is an essential part of all our lives. Following productivity increases in the Green Revolution, food prices in major world markets have been at a historical low in recent decades. Analysis from the Government Office for Science shows that by 2050, this situation is set to change significantly. The global population will have increased by nearly a third to nine billion, and diets will have changed with increasing affluence, leading to a much increased demand for food. At the same time, the food supply may be threatened as agriculture will have to compete with industry and municipal uses for energy and water. Climate change will also have adverse impacts on production in some areas.

The challenge is not only to increase food production, but to do so in a way that is sustainable, reducing our greenhouse gas emissions and preserving biodiversity. In addition, as demonstrated by the 2006–2008 food price spike, we must make the food system more resilient to volatility, both economic and climatic. A further challenge is that of ending hunger; currently around 1 billion people are hungry, and we must work to ensure that this number decreases rather than increases in the future.

Meeting these challenges will involve social and political solutions as well as those based on the natural sciences. The need for action is urgent given the time required for investment in research to deliver new technologies to those who need them, and for political and social change to take place. For this reason, the Government Office for Science has, with the assistance of leading stakeholders and experts, undertaken a Foresight study on the future of the global food and farming system. The papers presented in this special issue of *Philosophical Transactions of the Royal Society B* are reviews of the major drivers of change in the global food system looking out to 2050. They are intended to be accessible to experts in other fields.

While the individual papers are each important, they are of greatest value when brought together. Although a single journal edition cannot comprehensively cover the whole of the global food and farming system, I hope readers will find that the papers presented provide a significant and useful resource that attracts broad interest.

The Government Office for Science would welcome your response to this special issue. Any comments can be made via: www.foresight.gov.uk. The project's final report will be published on this website in late 2010.

